# Enhanced Electrocatalytic Performance of P-Doped MoS_2_/rGO Composites for Hydrogen Evolution Reactions

**DOI:** 10.3390/molecules30061205

**Published:** 2025-03-07

**Authors:** Wenjun Zhu, Bofeng Zhang, Yao Yang, Minghai Zhao, Yuwen Fang, Yang Cui, Jian Tian

**Affiliations:** 1 School of Mechanical and Electrical Engineering, Jingdezhen Ceramic University, Jingdezhen 333403, China; 2 Jingdezhen Mingxing Aerospace Forging Co., Ltd., Jingdezhen 333403, China; 3 Richangsheng Architectural New Materials Design Research lnstitute Co., Ltd., Hangzhou 310000, China; 4 School of Materials Science and Engineering, College of Chemical and Biological Engineering, Shandong University of Science and Technology, Qingdao 266590, China

**Keywords:** MoS_2_, P-doped, rGO, HER, alkaline condition

## Abstract

This study is based on the strategies of composite and element doping. Herein, P-MoS_2_/rGO materials were synthesized using a solvent-assisted hydrothermal method. The MoS_2_ nanosheets were uniformly and vertically grown on rGO; meanwhile, the optimized structure of MoS_2_ was achieved by P doping, resulting in improved catalytic performance and structural stability. Under alkaline conditions, the P-MoS_2_/rGO catalyst exhibits good electrocatalytic activity, demonstrating a Tafel slope of 70.7 mV dec^−1^ and an overpotential of 172.8 mV at 10 mA/cm^2^. Notably, even after 3000 consecutive LSV tests, the curves still show a high degree of overlap, indicating exceptional stability.

## 1. Introduction

As global energy demands surge and concerns over climate change escalate, transitioning from fossil fuels to sustainable energy sources has become critical [1,2,3]. Hydrogen, known for its clean combustion that generates only water as a byproduct, presents a promising alternative for mitigating greenhouse gas emissions typically associated with conventional energy sources [4,5,6]. Conventional hydrogen production techniques, including steam reforming and coal gasification, are frequently associated with high carbon emissions and energy consumption. In contrast, the hydrogen evolution reaction (HER) through water electrolysis enables the production of clean hydrogen, positioning it as a key direction in the green energy transition [7,8].

The advancement of efficient catalysts is essential for facilitating hydrogen production through the HER. In acidic HERs, protons (H^+^) are readily available in the electrolyte, enabling a direct electron transfer step (H^+^ + e^−^ → H*) to form adsorbed hydrogen (H^*^). In contrast, in alkaline HER, the reaction requires an additional water dissociation step (H_2_O + e^−^ → H^*^ + OH^−^) to generate protons before the electron transfer can occur. This extra step makes the alkaline HER significantly slower and more energy-intensive due to the need to break the strong H-O bond in water [9,10,11]. This fundamental difference underscores the need for advanced catalyst design to overcome the kinetic limitations of HERs in alkaline environments [12]. Conventional precious metal catalysts, like platinum and palladium, demonstrate excellent catalytic performance in electrocatalytic reactions under alkaline conditions; however, they face issues such as high cost and resource scarcity during long-term use. In contrast, molybdenum disulfide (MoS_2_) has garnered significant attention due to its excellent catalytic properties, stability in corrosive environments, and natural abundance [13,14,15]. The active sites of MoS_2_, primarily located at the edges, are crucial for HER efficiency, leading researchers to explore various strategies to maximize their availability. Recent research has primarily focused on modifying the morphology of MoS_2_ to increase its surface area and the density of active sites [16,17,18]. Despite some progress, MoS_2_ tends to aggregate during the reaction process and has poor conductivity, which results in suboptimal catalytic performance and stability. The composite design with high-conductivity materials, such as graphene or reduced graphene oxide, effectively enhances the material’s conductivity, promoting electron transfer and improving electrocatalytic performance. In addition, element doping is an effective method to significantly improve HER activity, as doping not only enhances the morphology of MoS_2_ but also activates its inert basal plane, resulting in the increased active sites and thereby improved HER performance. For instance, by introducing phosphorus (P) atoms into the MoS_2_ lattice, the number of active sites can be significantly increased, and the electrical conductivity can be improved, thereby enhancing its performance in the electrocatalytic HER. Typically, phosphorus doping needs to be achieved through complex chemical vapor deposition (CVD) or high-temperature annealing processes. These methods often require precise control of the reaction conditions and a relatively long processing time, which increases the complexity and cost of preparation [19,20,21].

**Figure 1:**
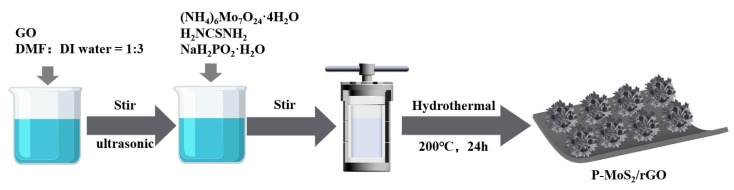
The synthetic process of P-MoS_2_/rGO composite.

Therefore, to improve catalytic activity and structural stability, P-MoS_2_/rGO composites were designed and fabricated using a straightforward one-step hydrothermal approach. The morphology and microstructure of MoS_2_ were optimized by P doping, and the P-doped MoS_2_ (with enlarged interlayer spacing and well dispersion, etc.) uniformly grew on the surface of rGO, improving the conductivity and the number of active sites as well as reducing the aggregation. Consequently, the P-MoS_2_/rGO composite demonstrates excellent HER performance in an alkaline (1.0 M KOH) solution (Figure 1).

## 2. Results and Discussion

**Figure 2:**
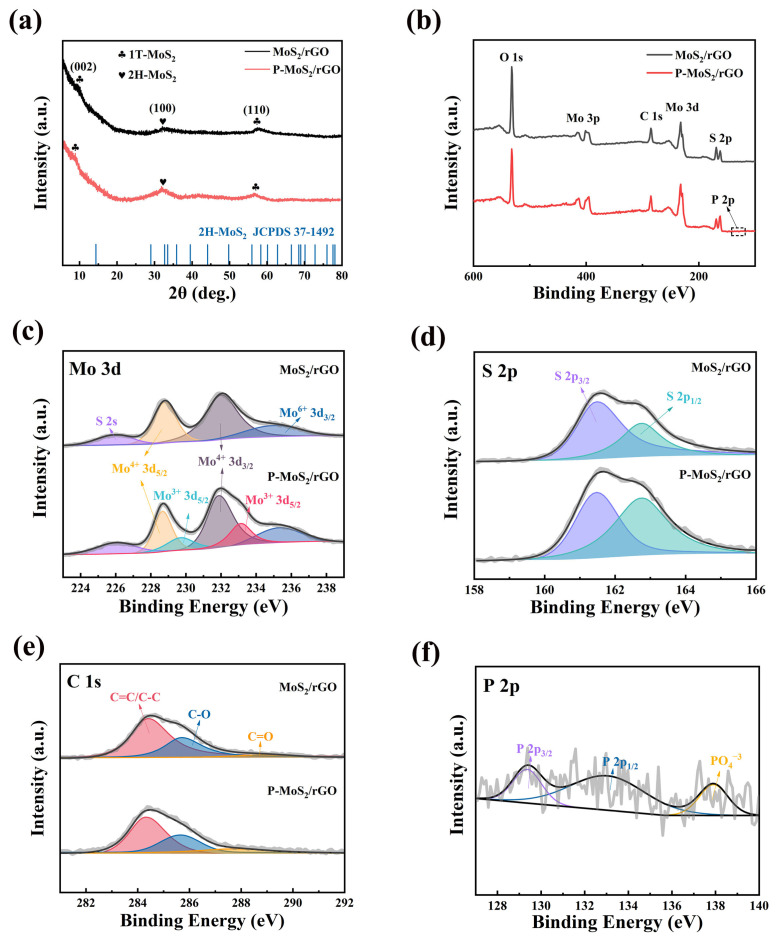
Chemical components of MoS_2_/rGO and P-MoS_2_/rGO, (**a**) XRD pattern, (**b**) XPS survey spectrum, XPS results of (**c**) Mo 3d, (**d**) S 2p, (**e**) C 1s, and (**f**) P 2p, respectively.

As shown in Figure 2a, XRD analysis reveals that in the MoS_2_/rGO composite, prominent peaks are observed at 9.7° and 57.6°, corresponding to the (002) and (110) planes of 1T-MoS_2_, respectively. Furthermore, a peak at 32.6° corresponds to the (100) plane of 2H-MoS_2_ (JCPDS 37-1492) [22]. A similar XRD curve is detected from the P-MoS_2_/rGO composite, indicating that the introduction of P does not lead to the formation of new phases. Meanwhile, compared with the MoS_2_/rGO composite, the (002) peak of the P-MoS_2_/rGO composite shifts to a lower angle at 9°, which demonstrates that the enlarged interlayer spacing can be obtained after P doping. According to the Bragg’s law (2dsinθ = nλ), the interlayer spacing of the (002) crystal plane is calculated to change from 0.75 nm to 0.83 nm [23]. Notably, the XRD patterns for both samples lack any distinct peaks attributed to GO or rGO, which can be attributed to their low abundance or effective dispersion within the matrix [24]. Moreover, the XPS tests were undertaken, and the results are shown in Figure 2b–f. In the XPS survey (Figure 2b), elements such as O, Mo, C, S, and P can be detected in both the MoS_2_/rGO and P-MoS_2_/rGO composites. According to the XPS survey, the Mo:S molar ratios in MoS_2_/rGO and P-MoS_2_/rGO are approximately 1:2 and 1:1.8, respectively. Compared with MoS_2_/rGO, the increased Mo: S molar ratio in the P-MoS_2_/rGO composite is attributed to the fact that some S atoms in the MoS_2_ lattice are replaced by P [25]. Moreover, the P-doping process may also lead to the generation of local lattice distortions or defects, further reducing the stability of S and resulting in the loss of S [26]. In the high-resolution Mo 3d spectrum of MoS_2_/rGO (Figure 2c), peaks corresponding to S 2s (~225.9 eV), Mo^4+^ 3d_5/2_ (~228.9 eV), Mo^4+^ 3d_3/2_ (~231.9 eV), and Mo^6+^ 3d_3/2_ (~235.4 eV) can be observed. It is evident that new peaks corresponding to Mo^3+^ 3d_5/2_ (229.7 eV and 233.1 eV) are observed in the Mo spectrum of P-MoS_2_/rGO, which can be attributed to the interaction between Mo and the less electronegative doping P atoms [21]. From the S spectrum (Figure 2d), two peaks corresponding to S 2p_3/2_ and S 2p_1/2_ are detected, which are located at 161.6 and 162.7 eV. In Figure 2e, three main peaks are found in the C 1s spectrum, including C=C/C-C (~284.4 eV), C-O (~285.7 eV), and C=O (~288.7 eV). The C=C/C-C peak comes from the benzene framework in graphene, while the C-O and C=O peaks originate from the oxidation of graphene [27]. Figure 2f shows that the P 2p spectrum peaks at 137.9, 129.4, and 133.0 eV are attributed to PO_4_^3−^, P 2p_3/2_ and P 2p_1/2_, respectively, indicating the successful incorporation of P into MoS_2_/rGO. Based on the above characterization results, the successful preparation of the samples (MoS_2_/rGO and P-MoS_2_/rGO composite) is confirmed.

**Figure 3:**
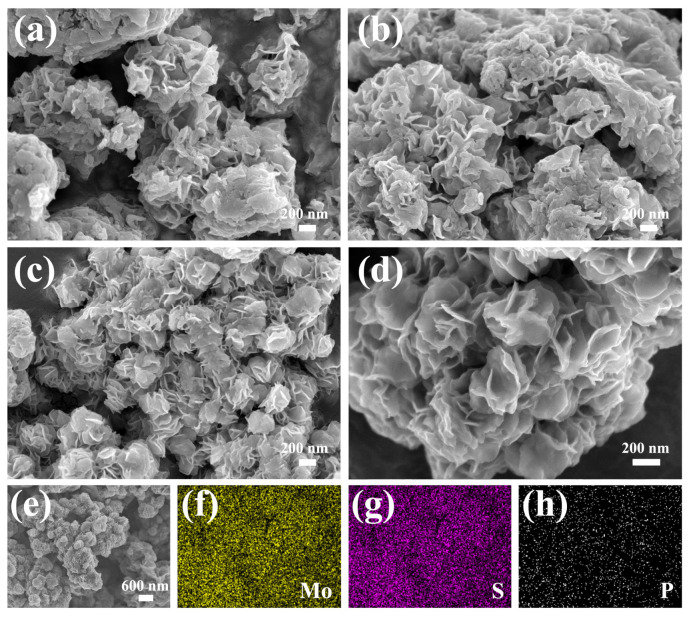
SEM results of the (**a**,**b**) MoS_2_/rGO, (**c**,**d**) P-MoS_2_/rGO, and (**e**–**h**) corresponding element mapping images of P-MoS_2_/rGO.

As presented in Figure 3a, unevenly distributed flower-like structures self-assembled from nanosheets can be found in the MoS_2_/rGO composite. Moreover, as displayed in the high-magnification SEM image (Figure 3b), there is significant aggregation among these nanosheets, and some blocks or granular materials (marked by) can be observed, which hinders the exposure of active sites. Compared with the MoS_2_/rGO composite, a similar flower-like structure, which is more evenly distributed and smaller in size (approximately 220 nm in diameter), can be detected from the P-MoS_2_/rGO composite (Figure 3c). Furthermore, as shown in Figure 3d, the flower-like structure of MoS_2_ is well formed (just like blooming flowers), and a porous structure can be acquired from the crosslinking effect of these nanosheets without interlayer aggregation, blocks, or granular materials, which is beneficial for providing more active sites [28]. Moreover, the different P-doping concentrations have an important effect on the morphology of the P-MoS_2_/rGO composites. As shown in Figure S1, significant aggregation among the nanosheets can be detected from P-MoS_2_/rGO-1 and P-MoS_2_/rGO-5 composites, which hinders the exposure of the active sites. Figure 3e–h present the EDS results, the uniform distribution of elements, especially the P element, and confirm the successful and uniform doping of P.

**Figure 4:**
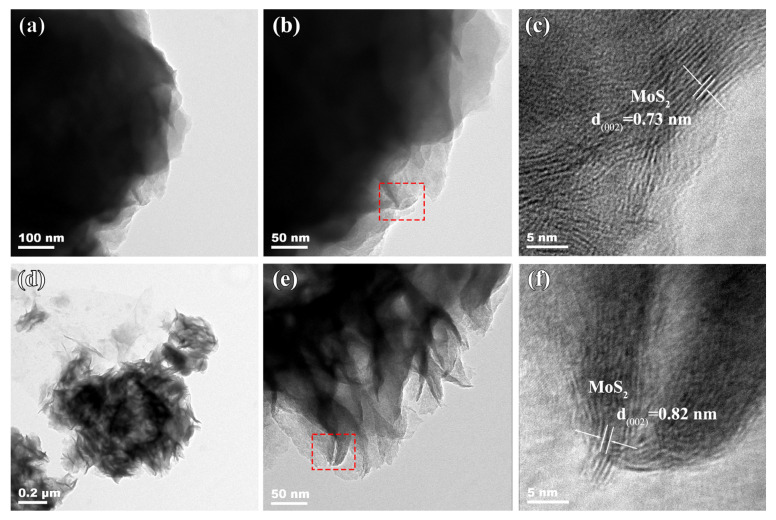
The TEM results of the (**a**–**c**) MoS_2_/rGO and (**d**–**f**) P-MoS_2_/rGO. (**c**,**f**) are the corresponding enlarged images of the red boxes in (**b**,**e**), respectively.

The microstructural characteristics of the composites were studied by TEM. As observed in the low-magnification TEM images in Figure 4a,b,d,e, MoS_2_/rGO exhibits numerous dark regions, whereas the P-MoS_2_/rGO shows more bright areas, indicating that the P-MoS_2_/rGO possesses a distinct porous structure derived from the co-crosslinking of nanosheets, aligning with the SEM results. As shown in the high-magnification images (Figure 4c,f), distinct lattice fringes with spacings of 0.73 nm and 0.82 nm are observed from both samples, corresponding to the (002) plane of 1T-MoS_2_. In accordance with the XRD results, the increased interlayer can be found in the P-MoS_2_/rGO composite, which is attributed to the P-doping effect. Furthermore, a comparison of Figure 4c,f shows that the lattice fringes in the P-MoS_2_/rGO composite exhibit distortion, indicating that the P doping has an effect on the microstructure (such as local defect, etc.); this in turn effectively improves electron transport and increases the catalytic activity of reaction sites [29].

**Figure 5:**
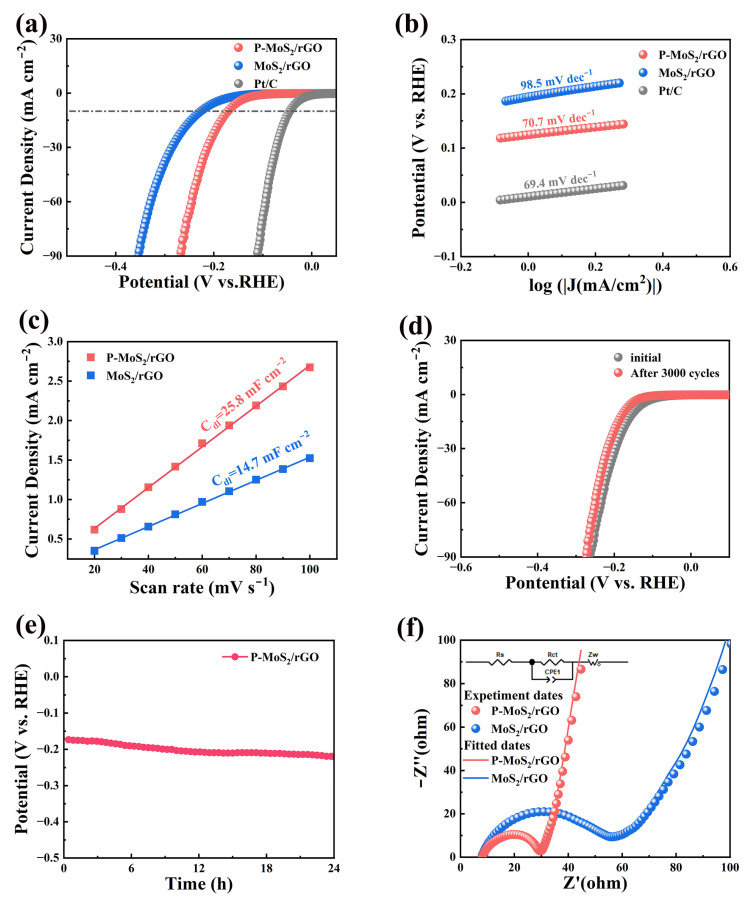
(**a**) Polarization curves, (**b**) Tafel slopes, and (**c**) C_dl_ values. Stability performance of P-MoS_2_/rGO material: (**d**) polarization curves and (**e**) time-dependent curves, (**f**) Nyquist plots.

The catalytic performance of the catalyst for HER was assessed, as shown in Figure 5. The linear sweep voltammetry (LSV) test (with a scan rate of 5 mV s^−1^) results are shown in Figure 5a with commercial Pt/C electrode for comparison. At 10 mA cm^−2^, the observed overpotentials of P-MoS_2_/rGO, MoS_2_/rGO, and Pt/C are 172.8, 228.4, and 44.7 mV, respectively, with corresponding Tafel slopes (Figure 5b) of 70.7, 98.5, and 69.4 mV dec^−1^, respectively. Compared with the MoS_2_/rGO composite, the P-MoS_2_/rGO composite displays superior HER performance with a lower overpotential and Tafel slope [30]. Moreover, as shown in Figure S2, the corresponding overpotentials of P-MoS_2_/rGO-1 and P-MoS_2_/rGO-5 composites are 205.2 mV and 230.5 mV, respectively. It is likely that a higher P concentration leads to excessive aggregation and phase instability, while a lower concentration fails to fully optimize the structure and electronic states, resulting in a decrease in the catalytic performance [31,32]. The electrochemical active surface area (ECSA) has an important effect on the HER performance, which can be evaluated in terms of double-layer capacitance (C_dl_). As shown in Figure 5c, the C_dl_ values were obtained according to the CV tests conducted at 20–100 mV s^−1^, and the corresponding values of both samples are 14.7 and 25.8 mF cm^−2^, respectively. That is to say, the improved electrochemical active sites can be obtained after P doping, which is attributed to the enlarged interlayer distance and optimized microstructure.

When evaluating the catalytic performance for HER, stability is a vital consideration. The LSV curves for the P-MoS_2_/rGO composite before and after 3000 potential cycles are presented in Figure 5d, which exhibit almost no change in shape, indicating a negligible decay in performance [33]. Moreover, the chrono-potentiometric test was conducted, as shown in Figure 5e. It can be found the potential remains stable over a 24 h period, demonstrating the excellent durability of the P-MoS_2_/rGO composite. EIS test was performed to further explain the superior HER performance, with fitting performed using an equivalent circuit model (inset), and the results are presented in Figure 5f. Compared with MoS_2_/rGO composite, the semicircle (R_ct_) with the smaller radius indicates an improved electron and charge transfer properties that can be achieved for the P-MoS_2_/rGO composite, and the corresponding R_ct_ values for both products are 22.3 and 59.6 Ω, respectively. Thus, the enhanced HER kinetics can be achieved for the P-MoS_2_/rGO composite [34]. Table 1 compares the catalytic performance of existing MoS_2_-based composites with our work. It can be observed that the P-MoS_2_/rGO composite in this work exhibits comparable catalytic performance, which can be attributed to the following several factors: Firstly, it is derived from the composite with rGO and P doping, resulting in improved conductivity. Furthermore, the optimized structure with enlarged interlayer spacing and well dispersion can be obtained by the P doping effect, providing abundant reaction sites and alleviating the stacking issue of MoS_2_. Notably, when the doping level of P in the P-MoS_2_/rGO composite material reaches the optimal state, it features a structure with a porous and uniform flower-like morphology. Thus, improved catalytic performance and structural stability can be achieved by the P-MoS_2_/rGO composite.

## 3. Experimental Section

### 3.1. Materials Synthesis

Reagents of analytical grade were used in this study without the need for further purification. As illustrated in Figure 1, 0.05 g of graphene oxide (GO, Suzhou Carbon Rich Graphene Technology Co., Ltd, Suzhou, China) was initially combined with 12 mL of deionized water and 4 mL of dimethylformamide (DMF, Shanghai Aladdin Biochemical Technology Co., Ltd, Shanghai, China), followed by stirring and ultrasonication for 1 h to achieve a uniform dispersion. Next, 0.4 g of thiourea (H_2_NCSNH_2_, Tianjin Fengchuan Chemical Technology Co., Ltd., Tianjin, China), 0.2 g of ammonium heptamolybdate tetrahydrate ((NH_4_)_6_Mo_7_O_24_·4H_2_O, Tianjin Damo Chemical Reagent Factory, Tianjin, China) and 0.03 g of Sodium hypophosphite monohydrate (NaH_2_PO_2_·H_2_O, Xilong Science Co., Ltd, Shantou, China) were added to the solution. A hydrothermal synthesis was then conducted at 180 °C for 24 h. The resulting product was subsequently ultrasonically washed to eliminate any organic contaminants and dried (60 °C for 18 h), obtaining the P-MoS_2_/rGO composites. In order to investigate the effect of different P-doping concentrations, the samples with the addition amounts of (NH_4_)_6_Mo_7_O_24_·4H_2_O being 0.01 g and 0.05 g were prepared for comparison, which were labeled as P-MoS_2_/rGO-1 and P-MoS_2_/rGO-5, respectively. For comparative purposes, MoS_2_/rGO was synthesized under the same conditions, omitting the addition of NaH_2_PO_2_·H_2_O.

### 3.2. Material Characterization

A powder X-Ray diffractometer (XRD; D8 Advance, Bruker AXS GmbH, Bellerica, MA, USA) equipped with Cu Kα radiation (λ = 0.15418 nm) was used to investigate the phase composition of the synthesized materials. The surface chemical composition and electronic states were studied using X-Ray photoelectron spectroscopy (XPS) on a Thermo Scientific K-Mura Alpha+ instrument (Thermo Scientific, Shanghai, China). To investigate the morphological features and microstructural properties, scanning electron microscopy (SEM; SU-8020, HITACHII, Beijing, China) and transmission electron microscopy (TEM; JEM-2100, JEOL, Beijing, China) were employed.

### 3.3. Electrochemical Measurements

A three-electrode device with a 1.0 M KOH electrolyte was used for the electrochemical measurements in which the Hg/HgO, glassy carbon electrode (4 mm in diameter) and graphite rod were used as the reference, working, and counter electrodes, respectively. All the results of cyclic voltammetry (CV), linear sweep voltammetry (LSV), current-voltage (I–V), and electrochemical impedance spectroscopy (EIS, 0.1 Hz–100 kHz) were recorded on an electrochemical workstation (CHI 660E, Chenhua, Shanghai, China). In the preparation process of the working electrode, 5 mg of the synthesized catalyst sample was dispersed in 1 mL mixture of 5% Nafion and anhydrous ethanol (with a volume ratio of 1:49). Following 2 h of ultrasonication, 5.0 μL of the catalyst suspension was evenly spread on the surface of the working electrodes and dried at room temperature for subsequent test. During the electrochemical measurements, the potentials were calibrated with reversible hydrogen electrode (RHE) according to the equation: E_RHE_ = E_Hg/HgO_ + 0.0592 × pH + 0.098.

## 4. Conclusions

In summary, this study presents a simple solvent-assisted hydrothermal method to synthesize P-MoS_2_/rGO materials. Based on the P-doping effect, the morphology and structure of MoS_2_ nanosheets were regulated, and the optimized MoS_2_ nanosheets (with enlarged interlayer spacing and well dispersion, etc.) were uniformly and vertically grown on rGO. Owing to the unique structure, improved conductivity, abundant reaction sites, and excellent structural stability can be achieved. As a result, the P-MoS_2_/rGO composite delivers superior HER electrocatalytic performance and cyclic stability, with an overpotential of 172.8 mV and a Tafel slope of 70.7 mV dec^−1^ at 10 mA/cm^2^. This work offers a simpler and more scalable synthesis route, while achieving comparable catalytic performance under alkaline conditions. These findings highlight the potential of P-MoS_2_/rGO composites as efficient and durable electrocatalysts for hydrogen production.

## Figures and Tables

**Table 1:** Compared with the existing MoS_2_-based composites.

Materials	Electrolyte	η_10_ (mV)	Tafel Slope (mV dec^−1^)	Refs
MoS_2_/SN-rGO	0.5 M H_2_SO_4_	650	184	[35]
MoS_2_-500	0.5 M H_2_SO_4_	355	84	[36]
M1S1	0.5 M H_2_SO_4_	248	84	[37]
rGO-MoS_2_	0.5 M H_2_SO_4_	207	-	[38]
Mn-MoS_2_/rGO	0.5 M H_2_SO_4_	230	76	[39]
500-MoS_2_	0.5 M H_2_SO_4_	180	47	[40]
MoS_2_/TiO_2_	1 M KOH	700	60	[41]
0.2 GO-MoS_2_ NIR	1 M KOH	314	80	[42]
Co_3_O_4_/MoS_2_	1 M KOH	205	128	[43]
MoS_2_/G HS	1 M KOH	183	127	[44]
MoS_2_/Ni_3_S_2_	1 M KOH	190	65.6	[45]
P-MoS_2_/rGO	1 M KOH	172.8	70.7	This work

## Data Availability

Data are contained within the article and Supplementary Materials.

## Supplementary Materials

The following supporting information can be downloaded at: https://www.mdpi.com/article/10.3390/molecules30061205/s1, Figure S1: SEM results of (a) P-MoS_2_/rGO-1 and (b) P-MoS_2_/rGO-5; Figure S2: Polarization curves.
