# Supplementary material for: Enhanced Electrocatalytic Performance of P-Doped MoS2/rGO Composites for Hydrogen Evolution Reactions
Source: Molecules. 2025 Mar 7;30(6):1205. doi: 10.3390/molecules30061205 (PMC11944752; doi:10.3390/molecules30061205)
Supplement: Supplementary file 1 [file molecules-30-01205-s001.zip › molecules-3451732-supplementary.pdf]

# Supporting information

## Enhanced Electrocatalytic Performance of P-Doped $MoS_2$/rGO Composites for Hydrogen Evolution Reactions

**Wenjun Zhu [1,2,3,*], Bofeng Zhang [1], Yao Yang [2], Minghai Zhao [1], Yuwen Fang [1], Yang Cui [3] and Jian Tian [4,*]**

[1] School of Mechanical and Electrical Engineering, Jingdezhen Ceramic University, Jingdezhen 333403, China
[2] Jingdezhen Mingxing Aerospace Forging Co., Ltd., Jingdezhen 333403, China
[3] Richangsheng Architectural New Materials Design Research Institute Co., Ltd., Hangzhou 310000, China
[4] School of Materials Science and Engineering, College of Chemical and Biological Engineering, Shandong University of Science and Technology, Qingdao 266590, China
* Correspondence: zhuwj@zju.edu.cn or zwj2730038@126.com (W.Z.); jiantian@sdust.edu.cn (J.T.)

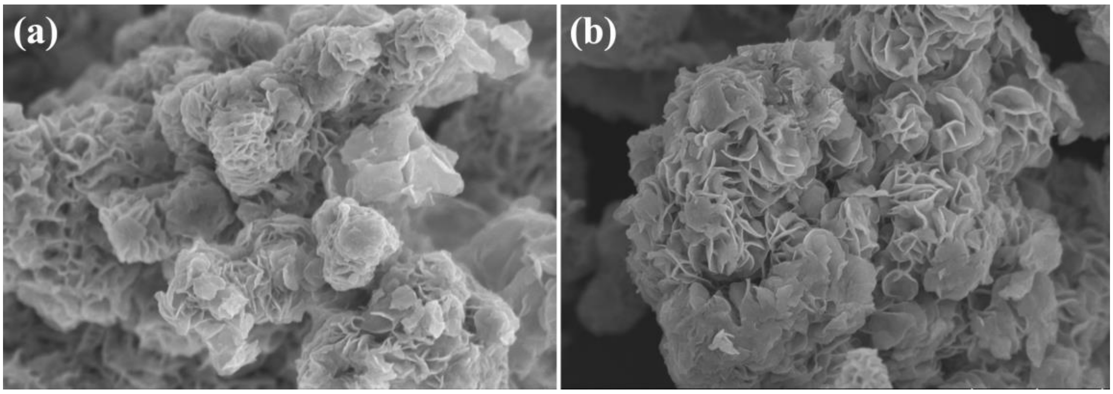


**Figure S1.** SEM results of (a) P-$MoS_2$/rGO-1 and (b) P-$MoS_2$/rGO-5.

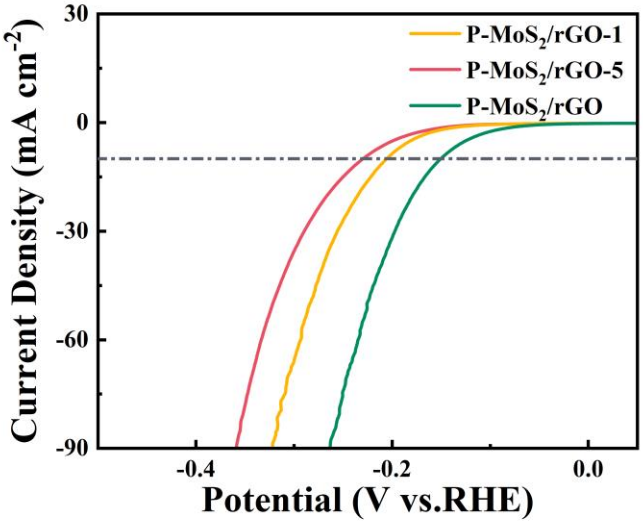


**Figure S2.** Polarization curves.
